# A 29-year-old man presented with acute upper gastrointestinal bleeding 

**Published:** 2020

**Authors:** Shabnam Shahrokh, Mohammad Pishgahi, Pardis Ketabi Moghadam, Fatemeh Naderi Noukabadi, Mohammad Reza Abdehagh, Asghar Arab Hosseini

**Affiliations:** 1 *Gastroenterology and Liver Diseases Research Center, Research Institute for Gastroenterology and Liver Diseases, Shahid Beheshti University of Medical Sciences, Tehran, Iran*; 2 *Basic and Molecular Epidemiology of Gastrointestinal Disorders Research Center, Research Institute for Gastroenterology and Liver Diseases, Shahid Beheshti University of Medical Sciences, Tehran, Iran *

## Question

**Figure 1:**
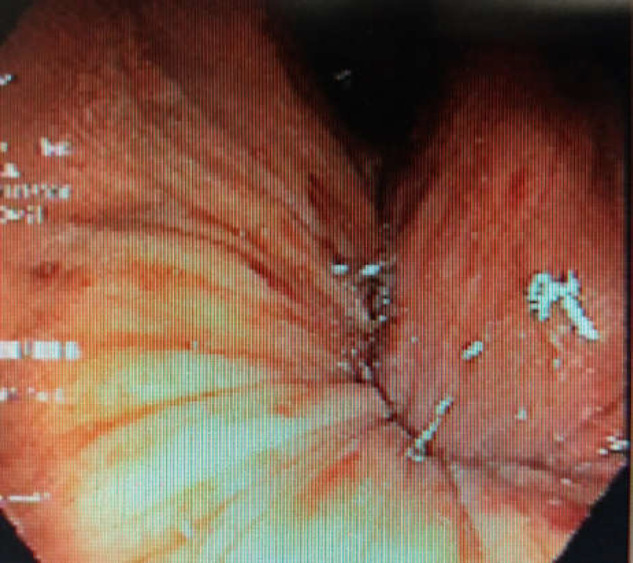
Retroflex view of the fundus is presenting an inflamed mucosa unable to be inflated

**Figure. 2:**
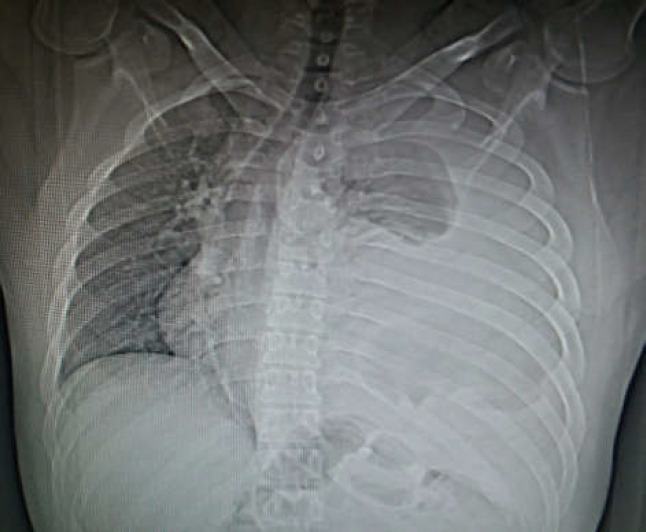
chest x ray

**Figure 3:**
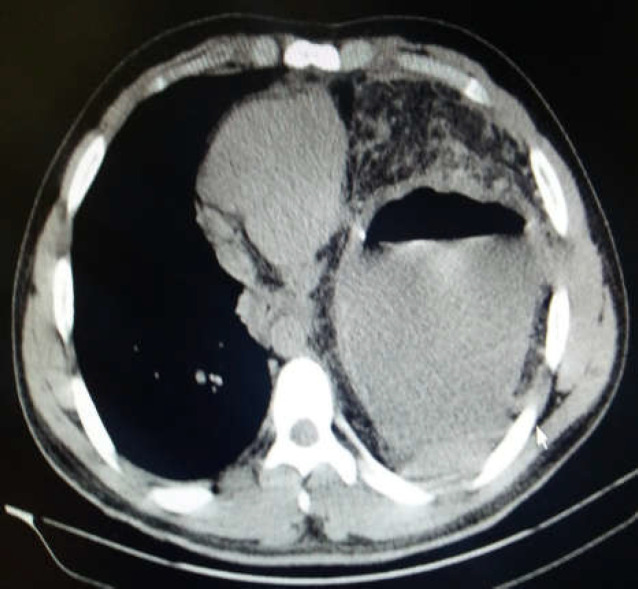
Chest spiral CT scan

 A 29-year-old man was admitted to the emergency department of Taleghani hospital; a teaching referral hospital in Tehran, Iran, with an acute upper gastrointestinal (GI) bleeding. On admission, he was found to have severe hematemesis since 6 hours before his arrival following an episode of intractable nausea and vomiting. He was single, residing in Tehran, Iran. He had a history of a 10 pack-year smoking, opium addiction and social alcohol consumption but denied usage of other illicit drugs, other medications including NSAIDs and other over-the-counter drugs. He had a history of stab wound 5 years ago leading to pneumohemothorax requiring a chest tube insertion. He did not mention any melena, defecation and gas passing since 2 days ago. He was not found to have any significant abdominal pain on admission. Four hours post admission, he was still conscious and oriented to the questions we were asking and he was still complaining about severe nausea. He had a temperature of 37.8o C. His blood pressure was 110/75mmHg. He had tachycardia of about 100 beats per minute. Resuscitation with IV fluids started. His physical examination was notable for a mild tenderness in left upper quadrant of the abdomen, dullness to percussion in the left thorax and decreased lung sounds on auscultation in the left lung but the examination of other organs was unremarkable. NG (nasogastric) tube was inserted and fresh bleeding was detected in the tube. His laboratory tests revealed an Hb of 12 gr/dl which was significantly lower than his previous Hb which was documented 18 gr/dl. No leukocytosis and thrombocytopenia was detected. His VBG was within normal limits. Kidney function tests, serum electrolytes, liver function tests, pancreatic enzymes and other biochemical tests were all unremarkable. Being hemodynamically stable but with an ongoing blood loss which demonstrated a serious risk for making him unstable in the near future, an emergent upper GI endoscopy was performed which revealed a completely congested and inflamed mucosa of fundus unable to be inflated in retroflex view. No active bleeding and source of bleeding was detected. Scope could not be passed into the distal part of stomach (figure-1). Abnormal auscultation of the lungs urged evaluation of thoracic field. Chest x ray (figure-2) and thoracic spiral CT scan (figure-3) were demanded. Six hours post admission, he was found to have tachypnea and shortness of breath. He spiked a heart rate of 120. 

**What is your diagnosis?**

**What is the next step?**

## Discussion

Traumatic diaphragmatic hernia is the herniation of abdominal organs into the chest cavity accompanied by a blunt and more common penetrating injury to the diaphragm (1). 

These injuries are driven to be repaired by surgical intervention to prevent a catastrophic herniation of abdominal organs and viscera to the thorax. If these injuries are left untreated, they would be presented with one of the most overwhelming complications of herniation which is called strangulated diaphragmatic hernia. This phenomenon is fatal and prompts an urgent surgery (2). 

**Figure 4:**
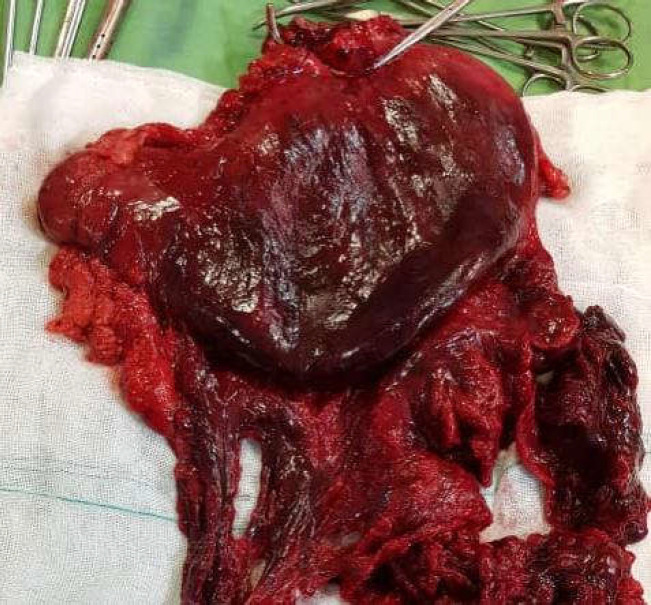
Total gastrectomy of strangulated stomach which shows a complete gangrene of the stomach

Our patient had a history of penetrating trauma to the left thorax requiring a chest tube insertion. He denied any other follow-up after the removal of his chest tube and it seems that the rupture of the left diaphragm had been neglected in this patient. The common symptom of a strangulated diaphragmatic hernia is a dramatic sudden severe upper abdominal or lower thoracic pain radiating to shoulder and neck, which was initially absent in our patient due possibly to the usage of opioids masking his pain (2). Other foreseeable symptoms are nausea, vomiting and GI bleeding (3) which were dominant in our patient. Besides, he experienced symptoms related to the involvement of chest cavity. Dyspnea and cyanosis are the symptoms reported as a result of mediastinal shift due to massive herniation of abdominal viscera into the thorax (4, 5). On admission, our patient was not found to have severe dyspnea or decreased O2 saturation although there was a prominent mediastinal shift into the right. Gradually, he was found to have difficulty in breathing leading to tachypnea and tachycardia. His spiral chest CT scan revealed a large air-fluid level in the left hemithorax indicating herniation of stomach into the left thorax. A surgery consultation was demanded. He was started on broad spectrum antibiotics and finally transferred to the operation room for an urgent surgery with the clinical scenario suggestive of a strangulated diaphragmatic hernia. Because of a complete gangrene of the strangulated stomach (figure 4), the patient went on a total gastrectomy and roux-en-y esophagojejunostomy with diaphragmatic defect repair. About 2 liters of serosangenous fluid was detected in the left hemithorax which was drained. After the surgery, the patient recovered more rapidly than expected and transferred to the general ward with precautions.
